# Food Polyamine and Cardiovascular Disease -An Epidemiological Study-

**DOI:** 10.5539/gjhs.v4n6p170

**Published:** 2012-09-28

**Authors:** Kuniyasu Soda, Yoshihiko Kano, Fumihiro Chiba

**Affiliations:** 1 Department of Cardiovascular Research Institute, Saitama Medical Center, Jichi Medical University, Saitama, Japan; 2 Department of Surgery, Saitama Medical Center, Jichi Medical University, Saitama, Japan

**Keywords:** polyamine, spermine, spermidine, cardiovascular diseases, Mediterranean diet

## Abstract

The purpose of this study was to examine the contribution of dietary polyamines toward preventing cardiovascular disease (CVD). Age-standardized mortality rates as well as other relevant information regarding individuals with CVD were gathered from the World Health Organization and the International Monetary Fund in 48 different European and other Western countries. Food supply data were collected from the database of the United Nations, and the amount of dietary polyamines was estimated by using polyamine concentrations in foods from published sources. The association between CVD mortality and the amount of polyamines was investigated by performing a series of multiple linear regression analyses. Analyses using factors known to modulate the risk of CVD including: Gross Domestic Product (GDP) (standardized regression coefficient (r) = -0.786, *p* < 0.001) and the amount of fruits, vegetable, nuts, and beans (r = -0.183, *p* = 0.001) but not including polyamines, showed negative associations with CVD, while smoking rate (r = 0.139, *p* = 0.041) and whole milk amount (r = 0.131, *p* = 0.028) showed positive associations with CVD. When the amount of polyamines was added to the analyses as a covariate, GDP (r = -0.864, *p* < 0.001) and polyamines (r = -0.355, *p* = 0.007) showed negative associations with CVD, while smoking rate (r = 0.183, *p* = 0.006) and whole milk (r = 0.113, *p* = 0.041) showed positive associations with CVD. The inverse association between dietary polyamines and CVD mortality revealed by the present study merits further evaluation.

## 1. Introduction

The prevalence of cardiovascular disease (CVD), one of the consequences of atherosclerosis, increases with age and is the leading cause of morbidity and mortality in industrialized nations. CVD incidence is also rapidly increasing in developing countries. In addition to life style, body weight, socio-economic environment, and certain pre-existing conditions, a number of foods seem to play a role in the incidence of CVD (Daviglus et al., 1997; Ignarro, Balestrieri, & Napoli, 2007).

Although the exact components in these foods and the mechanisms by which they contribute to preventing CVD are not fully understood, recent studies have shown the importance of inflammation in the mechanism of atherosclerosis and CVD (Mozaffarian, 2006; Nettleton et al., 2006). Some food components with anti-inflammatory properties may decrease risk of CVD and *vice versa* (Calder, 2006; Kinsella, Lokesh, & Stone, 1990; Mozaffarian, 2006; Nettleton et al., 2006). We have found that the natural polyamines (spermine and spermidine), which many foods contain in wide-ranging concentrations, suppress the synthesis of pro-inflammatory cytokines by peripheral blood mononuclear cells following *in vitro* lipopolysaccharide stimulation, as well as decrease the expression of leukocyte function-associated antigen 1 (LFA-1) on non-stimulated peripheral human blood mononuclear cells *in vivo* (Soda et al., 2005; Zhang et al., 1997). Polyamines are absorbed from the intestinal lumen and distributed to organs and tissues (Bardócz, Grant, Brown, Ralph, & Pusztai, 1993), and long-term consumption of polyamine-rich foods increases steady-state blood polyamine concentrations in mice and in humans (Soda, Kano, Takao, Lefor, & Konishi, 2009). Because almost all of the blood polyamines are associated with blood cells, the blood polyamine concentrations change in parallel with those in white blood cells. Therefore, changes in blood polyamine, in turn, affect cellular immune activities associated with LFA-1 function (Cohen, Lundgren, & Farrell, 1976; Cooper, Shukla, & Rennert, 1978; Kano et al., 2007). These findings prompted us to test the possible effects of food polyamines on age-associated diseases and longevity of vertebrates, and we have shown that long-term intake of polyamine-rich foods extends longevity of mice by decreasing incidence of age-associated pathologies, notably glomerulosclerosis, an age-related condition linked to atherosclerosis in humans (Kasiske, 1987; Soda, Dobashi, Kano, Tsujinaka, & Konishi, 2009).

Therefore the present study was designed to examine the statistical association between the amount of dietary polyamine and the mortality rate associated with CVD, as a representative of age-associated diseases, using several public database resources

## 2. Methods and Materials

### 2.1 Data Base

Dietary data were gathered from the online database of the Statistics Division of the Food and Agriculture Organization of the United Nations (FAOSTAT) and levels of food supply in 2002 were used. Since seafood supply data for Belgium in 2002 were unavailable, 1999 data were used because they were the most recent. The age-standardized mortality rates due to cardiovascular diseases (CVD) (per 100,000 population) in 2002 and male and female life expectancies at birth in 2000 were derived from the World Health Organization Statistical Information System (WHOSIS). The target populations included 49 countries in Europe, North America, and Oceania with similar racial and ethnic composition and social and religious backgrounds. Albania, Armenia, Australia, Austria, Azerbaijan, Belarus, Belgium, Bosnia and Herzegovina, Bulgaria, Canada, Croatia, Cyprus, Czech Republic, Denmark, Estonia, Finland, France, Georgia, Germany, Greece, Hungary, Iceland, Ireland, Israel, Italy, Kazakhstan, Latvia, Lithuania, Malta, Netherlands, New Zealand, Norway, Poland, Portugal, Romania, Russian Federation, Slovakia, Slovenia, Spain, Sweden, Switzerland, Tajikistan, The former Yugoslav Republic of Macedonia, Turkey, Turkmenistan, Ukraine, United Kingdom, United States of America, and Uzbekistan were surveyed.

The rate of smoking in Tajikistan was not available so data were gathered from the other 48 European countries. The rate of male and female obesity [body mass index (BMI) greater than 30] in 2001 and the rate of tobacco use among males and females over 15 years of age between 1996 and 2003 among these 48 countries were gathered from WHOSIS. Since rates of obesity and tobacco use were not found for each gender, the whole population rates for obesity and tobacco use were used instead. Per capita pure alcohol supply among adults (over 15 years of age) in 2003 was also obtained from WHOSIS. In order to gauge the role of socio-economic factors, Gross Domestic Product (GDP) (PPP: purchasing power parity) per person in 2002 was obtained from the International Monetary Fund (IMF).

Concentrations of polyamines, spermine and spermidine in foods were from published reports of concentrations measured in European foods (Bardócz, Grant, Brown, Ralph, & Pusztai, 1993; Cipolla, Havouis, & Moulinoux, 2007). When these reports did not show polyamine concentrations for specific foods, or additional data were necessary to obtain an accurate average concentration in a food, we used data from Nishibori et.al. (2007) (Table 1).

**Table 1:** Concentrations of polyamines in foods (nmol/g or nmol/mL)^1^

	spermine	spermidine
Apple^2^	0	14.73
Banana	1.00	44.90
Lemon & lime	0.90	18.40
Citrus (other)	0.90	18.40
Pineapple	10.90	27.00
Grape^3^	1.60	22.50
Orange & mandarin^4^	0	41.40
Other fruits^5^	3.02	25.50
Pulses^6^	66.46	179.7
Treenuts^7^	46.93	186.97
Groundnut	34.60	388.70
Cereals^8^	17.94	57.55
Potato^9^	7.90	64.70
Maize^10^	8.00	144.00
Onion^11^	2.50	41.20
Tomato^12^	0	19.35
Vegetables^13^	6.69	124.13
Stimulants^14^	12.50	61.40
Oil crops	0	0
Sugar	0	0
Coffee	0	0
Alcoholic beverages^15^	0	1.00
Beer^16^	0	0.50
Wine^17^	0	2.17
Animal fats	0	0
Beef^18^	120.7	22.45
Butter & Ghee	0	0.5
Cephalopods^19^	86.00	13.50
Cheese^20^	21.581	145.337
Cream	0	0
Crustaceans^21^	0	1.98
Edible offals^22^	98.90	82.28
Eggs	0	0
Fish^23^	16.25	16.35
Honey	0	1.00
Meat^24^	110.53	29.68
Molluscs^25^	94.43	73.13
Mutton & Goat meat	131.30	39.70
Other Marine meat^26^	37.76	25.46
Pork	160.15	18.15
Poultry^27^	91.70	27.50
Whey^28^	1.00	1.00
Whole Milk	0	0

1 For the polyamine concentrations in each food, the mean concentrations in the following foods were used:

2 Jonagold, Golden, and Granny Smith,

3 Red grape and green grape.

4 Orange and orange (Bardocz).

5 Raisin, prune, pear, peach, yellow peach, dates, kiwi, strawberry, and melon.

6 French bean, red bean, garden pea, soyabean (Bardocz), and red kidney bean (Bardocz).

7 Hazelnut, almond, and pistachio.

8 Rice, semolina, pasta, white bread, oat bread, rye bread, and whole wheat bread.

9 Potato, skinned; potato with skin; and potato (Bardocz).

10 Maize (Nishibori).

11 Onion and onion (Bardocz).

12 Tomato and tomato (Bardocz).

13 Salsify, celery, carrot, green cabbage, beet, beetroot, carrot, sorrel, radish, chicory, leek, escarole, red cabbage, green leek, Brussels sprout, lettuce, chervil, cabbage, parsley, mushroom, and button mushroom.

14 Garlic, yellow pepper, green pepper, and red pepper.

15 Whisky and Cognac.

16 Lager beer, and stout beer.

17 White (Burgundy), white (Loire), red (Bordeaux), red (Cotes-du-Rhone), red (Touraine), and red (Beaujolais) wines.

18 Veal and beef.

19 Squid and octopus (Nishibori).

20 Soft cheese, Swiss Emmental, French Emmental, goat cheese without rind, Brie pasteurized without rind, graded cheese, Camembert, Brie pasteurized with rind, goat cheese with rind, Roquefort, sweet Cantal with rind, Comte, Saint Nectaire without rind, Saint Nectaire with rind, aged cheddar (Bardocz), and fresh cheddar (Bardocz).

21 Scampi, shrimp, crayfish, and crab claw.

22 Ox tongue, liver mousse, chitterling, duck liver paste, and pork liver paste.

23 Hake, cod, whiting, smoked salmon, mullet, fresh salmon, cod (Bardocz), and trout (Bardocz).

24 Veal, pork, turkey, chicken leg, rabbit, lamb, chicken wing, and beef.

25 Oyster, white scallop, coral scallop, and clam (Nishibori).

26 Hake, cod, whiting, smoked salmon, mullet, fresh salmon, cod (Bardocz), trout (Bardocz), scampi, shrimp, crayfish, crab, squid, octopus (Nishibori), oyster, white scallop, coral scallop, and clam (Nishibori).

27 Turkey wing, chicken leg, and chicken wing.

28 No available data, therefore data of matured yogurt were used. Concentrations of polyamines in foods with no superscript indicate that they were from a single food.

Polyamine concentrations were expressed as nmol/g or mL.

^23^The amount in fish was a sum of the amounts in freshwater fish, and demersal, pelagic, and other marine fish, and ^26^ the amount in other marine meat was obtained by subtracting the sum of the amounts in fresh water fish, demersal and pelagic fish, other marine fish, crustaceans, mollusks, and cephalopods from the amount in fish & seafood in the FOSTAT database. Aquatic animals and other aquatic products were not consumed in surveyed countries.

Polyamine concentrations in foods were taken from Cipolla et al. (2007). Polyamine contents in current foods: a basis for polyamine reduced diet and a study of its long term observance and tolerance in prostate carcinoma patients. *Amino Acids, 33*, 203-12. Those marked as (Bardocz) were from Bardocz et al. (1993). Polyamines in food-implications for growth and health. *J. Nutr. Biochem., 4*, 66-71, and (Nishibori), from Nishibori et al. (2007). Amounts of polyamines in foods in Japan and intake by Japanese. *Food Chem, 100*, 433-872.

### 2.2. Statistical Analysis

In addition to polyamine as a covariate, other relevant factors, used as independent variables, were selected based on previously published reports. Some of the obtained data were transformed to approximate a normal distribution. The dependent variable was the natural logarithm of CVD/capita/year and independent variables were as follows: Total caloric supplied (kcal/capita/day), total fat supplied (g/capita/day), amount of dietary polyamine (µmol/capita/day), whole milk supplied (g/capita/day), square root of all milk products including whole milk supplied (g/capita/day), natural logarithm of Vegetables etc (fruits, vegetable, nuts, and beans) supplied (g/capita/day), pure alcohol supplied (ml/capita/day), square root of mixed alcoholic drink supplied (g/capita/day), square root of wine supplied (g/capita/day), square root of the ratio of seafood to animal meat, square root of the rate of obesity, smoking rate, and square root of the GDP.

Associations between independent and dependent variables were tested by simple linear regression analysis. To assess the contribution of these independent variables to CVD mortality, we designed a multiple linear regression model with CVD mortality as the outcome or dependent variable. A *p* value of less than 0.05 was considered significant. All statistical analyses were performed using StatView 5.0 for Apple computers.

## 3. Results

### 3.1 Regression between CVD and Longevity

Simple regression analysis showed very strong negative correlations between CVD (using data without transformation) and male life expectancy at birth in 2000 (r = -0.944, p < 0.001) and between CVD and female life expectancy (r = -0.946, p < 0.001), indicating CVD as one of the most important factors that affect longevity in all studied countries.

### 3.2 Linear Regression Analysis between Independent Variables

As a first step, linear regression analyses between independent variables were performed (Table 2) in order to eliminate the possibility of multicolinearity among covariates. Fat was strongly correlated with caloric (r = 0.875), dairy products (r = 0.803), and GDP (r = 0.902). Since fat strongly affects caloric due to its high energy content, it was excluded from the multiple linear regression analyses. Vegetables etc had a close correlation with polyamine (r = 0.812); therefore, analyses were performed with or without Vegetables etc as an independent variable. Finally, all milk products had a close correlation with GDP (r = 0.834).

**Table 2:** Linear regression analyses

	Fat	PA	Whole milk	Dairy products	Veg. etc	Alcohol	S/M ratio	Obesity rate	Smoking rate	GDP
Calorie	0.876	0.745	-0.267	0.679	0.539	0.584	0.530	0.119	0.122	0.785
Fat	-	0.605	-0.360	0.803	0.497	0.608	0.447	0.084	-0.026	0.902
PA		-	-0.286	0.402	0.812	0.391	0.267	0.179	0.294	0.482
Whole milk			-	-0.522	-0.268	-0.203	-0.192	-0.216	-0.034	-0.340
Dairy products				-	0.312	0.503	0.525	0.093	-0.045	0.834
Veg. etc					-	0.119	0.157	0.290	0.091	0.419
Alcohol						-	0.336	0.013	0.283	0.585
S/M ratio							-	0.093	0.150	0.510
Obesity rate								-	0.048	0.140
Smoking rate									-	-0.141

Dairy products include all products from milk including whole milk. Veg. etc includes vegetables, fruits, nuts, and beans, but excludes potatoes. Alcohol is the sum of the amount of all alcoholic drinks. Some of the figures are transformed to approximate normal distribution.

PA = polyamine; Veg. = vegetables; S/M ratio = seafood/meat ratio.

### 3.3 Multiple Linear Regression Analyses using Independent Factors that had Previously Ascertained the Relation with CVD

Before testing the association between dietary polyamine and CVD, multiple linear regression analyses were performed using independent factors that had previously been shown to affect incidence of CVD, but not including polyamine. Among independent variables, GDP and Vegetables etc showed inverse associations with CVD (*p* < 0.05). Meanwhile, smoking rate and whole milk had positive correlations with CVD (*p* < 0.05). In addition, although the regression coefficient was small and not statistically significant, associations between other foods and CVD were similar to what had been previously reported, e.g. alcohol (ranging from 3.2 to 37.5 mL/capita/day) and a high ratio of seafood to meat were negatively correlated to CVD. In contrast, total caloric supply and obesity were positively correlated with CVD (Table 3-a). When the all milk products, instead of whole milk, was used in the analysis, all milk products showed a negative correlation to CVD. However, the regression trends for independent variables other than milk products were not changed (Table 3-b).

**Table 3:** Multiple linear regression analyses using independent factors previously shown to affect incidence of CVD

**a)** analysis with whole milk	Standard			95% confidence intervals

	regression coefficient	t value	*p* value	
Total calorie	0.037	0.359	0.72	-0.00025 to 0.00036
Whole milk	0.131	2.280	0.03	0.00006 to 0.001
Vegetables etc	-0.183	-2.587	0.01	-0.620 to -0.076
Alcohol	-0.019	-0.239	0.81	-0.010 to 0.008
Seafood: meat ratio	-0.023	-0.348	0.73	-0.376 to 0.265
Obesity rate	0.026	0.463	0.65	-0.067 to 0.107
Smoking rate	0.139	2.114	0.04	0.0005 to 0.021
GDP	-0.786	-7.296	<0.001	-0.013 to -0.007

*d* (Durbin-Watson)= 1.740				

**b)** analysis with dairy products	Standard			95% confidence intervals

	regression coefficient	t value	*p* value	
Total calorie	0.062	0.585	0.56	-0.00022 to 0.0004
Dairy products	-0.165	-1.634	0.11	-0.030 to 0.003
Vegetables etc	-0.213	-2.925	0.005	-0.682 to -0.124
Alcohol	-0.031	-0.377	0.71	-0.011 to 0.008
Seafood: meat ratio	-0.011	-0.153	0.88	-0.359 to 0.309
Obesity rate	0.005	0.084	0.93	-0.085 to 0.092
Smoking rate	0.142	2.086	0.04	0.0003 to 0.022
GDP	-0.697	-5.106	<0.001	-0.012 to -0.005

*d* (Durbin-Watson)= 2.048				

### 3.4 Multiple Linear Regression Analyses with Polyamine Intake

In a second step, polyamine was added as an independent variable, and multiple linear regression analyses were repeated. As shown in Table 4, GDP and polyamine had significantly negative associations with CVD, while smoking and whole milk had positive associations with CVD. The *d*-value of the Durbin–Watson statistical test used to detect the presence of autocorrelation in the residuals from a regression analysis was 1.859, still suggesting the possibility of positive serial correlation (Table 4a). Therefore, analyses were done eliminating specific fruits and vegetables that contained significant amounts of polyamines, and the results of the analyses showed that polyamine, when properly isolated, resulted in lower *p* values (r = -0.329, *p* = 0.0002). Calorie supplied was also removed from the list of independent variables (Table 4b), because calorie has a close association with obesity, and the analysis showed that, along with the GDP, polyamine sill had a trend of negative association with CVD.

**Table 4:** Multiple linear regression analyses

**a)** analysis with calorie	Standard			95% confidence intervals

	regression coefficient	t value	*p* value	
Polyamine	-0.355	-2.836	0.007	-0.016 to -0.003
Total calorie	0.231	1.965	0.06	-0.00001 to 0.001
Whole milk	0.113	2.120	0.04	0.00005 to 0.001
Vegetables etc	0.028	0.279	0.78	-0.328 to 0.432
Alcohol	0.019	0.259	0.80	-0.008 to 0.010
Seafood: meat ratio	-0.046	-0.743	0.46	-0.407 to 0.188
Obesity rate	0.012	0.227	0.82	-0.071 to 0.090
Smoking rate	0.183	2.923	0.006	0.004 to 0.024
GDP	-0.864	-8.396	<0.001	-0.013 to -0.008

*d* (Durbin-Watson)= 1.858				

**b)** analysis with calorie	Standard			95% confidence intervals

	regression coefficient	t value	*p* value	
Polyamine	-0.212	-2.009	0.05	-0.011 to 0.00004
Whole milk	0.127	2.326	0.03	0.00007 to 0.001
Vegetables etc	-0.012	-0.119	0.91	-0.408 to 0.362
Alcohol	0.031	0.412	0.68	-0.007 to 0.011
Seafood: meat ratio	-0.012	-0.192	0.85	-0.324 to 0.268
Obesity rate	0.010	0.181	0.86	-0.076 to 0.091
Smoking rate	0.180	2.776	0.008	0.004 to 0.024
GDP	-0.755	-8.405	<0.001	-0.012 to -0.007

*d* (Durbin-Watson)= 1.747				

### 3.5 Linear Regression Analysis between Polyamine Intake and Other Foods

Finally, correlations between dietary polyamines and foods previously established to modulate risk of CVD were tested. Dietary polyamines had a strong positive correlation with Vegetables etc (r = 0.812, *p* < 0.001), fruits and vegetables (excluding nuts and beans) (r = 0.808, *p* < 0.001), and nuts and beans (r = 0.715, *p* < 0.001), and, to a lesser extent, of all milk products (r = 0.402, *p* = 0.004), wine (r = 0.0423, *p* = 0.003), and alcohol (r = 0.391, *p* = 0.006). In spite of a positive correlation with all milk products, polyamine had a trend of negative correlation with whole milk (r = -0.286, *p* = 0.048).

## 4. Discussion

In this epidemiological study, increase in dietary polyamines (spermine and spermidine) was associated with lower incidence of CVD. The data used for this purpose had several limitations, including the inability to establish correlations on an individual basis. In addition, part of the data were qualitative in nature, i.e. the obesity rate indicated the rate of people whose BMI was over 30 but did not indicate the mean BMI in each country. Moreover, other factors relevant to CVD mortality rate, such as the proportions of individuals with hypertension and performing regular physical activity, could not be obtained. Despite these drawbacks, however, the present analyses using factors previously established to modulate incidence of CVD yielded results very similar to that of previous studies.

Tobacco use is widely accepted as a major risk factor for the development of CVD resulting from direct effects on atherosclerosis (“The Surgeon General’s 1989 Report on Reducing the Health Consequences of Smoking: 25 Years of Progress”, 1989). Similarly, this study showed smoking as one of the major factors with a positive association with CVD mortality. Obesity is associated with many risk factors for CVD, such as hypertension, dyslipidemia (increased triglycerides and reduced HDL), and insulin resistance (Romero-Corral et al., 2006). Since obesity results from an imbalance between energy expenditure and energy consumption, and since caloric restriction causes beneficial metabolic, hormonal, and functional changes, increased caloric intake which is often accompanied with increased fat intake, as observed in the present study, is considered one of the factors associated with increased CVD. In the present analyses, both food calorie and obesity rate had positive regression coefficient values with CVD.

Socioeconomic status, measured by education, occupation, and income, have been reported to affect overall and cause-specific mortality, such as CVD, for several populations (Howard, Anderson, Russell, Howard, & Burke, 2000; Mackenbach, Cavelaars, Kunst, & Groenhof, 2000). In this study, data for education and occupation were not available, so the GDP was used instead to represent socioeconomic status in each country. These analyses showed that GDP was one of the most important factors having a negative association with CVD mortality among countries.

In previous studies, consumption of whole milk has at times been shown to have a positive correlation with CVD, while cheese and fermented milk consumption have at times been shown to have an inverse association with CVD events (Artaud-Wild, Connor, Sexton, & Connor, 1993; Moss & Freed, 2003; Segall, 1977). The reasons for these results are not completely understood but this study showed consistent results. An increased intake of fruits, vegetables, nuts and beans, of seafood relative to meat, and moderate amount of alcohol, commonly referred to as the “Mediterranean diet”, all shown to be associated with decreased CVD events, were likewise negatively associated with CVD mortality in this study (Keys, 1995; Mukamal et al., 2003; Trichopoulou, 2004). Alcohol consumption in the data used for analysis was within a so-called moderate range. Although a Mediterranean diet is associated with decreased CVD and prolonged longevity, exact nutrients and mechanisms by which these foods protect from CVD events are not necessarily well understood (Sacks et al., 2006). Interestingly, dietary polyamines had positive correlations with all of the foods that have negative associations with CVD. These indicate the possibility that dietary polyamines may have been a confounding factor in previous reports.

The biological mechanism by which polyamines prevent CVD should be further investigated. Of note, the results of this study were consistent with preceding animal experiments in which increased polyamine intake decreased the incidence of age-associated pathologies and mortality in aged mice. Polyamine is indispensable not only for cell growth and differentiation but also for cell function. In addition, polyamine from extracellular sources exerts strong anti-inflammatory properties (Soda et al., 2005; Soda, Kano, Takao, Lefor, & Konishi, 2009). N-3 PUFAs with anti-inflammatory properties and lipid lowering drugs such as statins with a LFA-1 inhibitory function have been shown to prevent CVD events (Levantesi et al., 2007; Weitz-Schmidt, 2003). Therefore, it is likely that the immunological and biological activities of polyamine exert similar effects as n-3 PUFAs and statins. In view of the role inflammation may play in age-associated diseases (Franceschi et al., 2000; Kinlay & Egido, 2006), natural anti-inflammatory substances such as polyamines and n-3 PUFAs may offer keys to human health and longevity.

Increases in polyamine concentrations in the body are closely related to neoplastic growth (Russell, 1983). Many studies have shown that increased polyamine synthesis and/or increased polyamine intake accelerates tumor growth, and conversely, the inhibition of polyamine synthesis and/or restriction of polyamine intake decelerates tumor growth (Clifford, Morgan, Yuspa, Soler, & Gilmour, 1995; Hibshoosh, Johnson, & Weinstein, 1991; O’Brien, Megosh, Gilliard, & Soler, 1997; Quemener et al., 1994; Sarhan, Knodgen, & Seiler, 1992). However, most of these studies were conducted to test the effects of polyamines on existing tumors or on the growth of tumors after neoplastic transformation or exposure to cancer initiators. It was shown that oncogenesis does not occur when normal cells are targeted for overexpression of enzymatic activities for polyamine synthesis and a resulting increase in polyamine concentration is observed (Clifford, Morgan, Yuspa, Soler, & Gilmour, 1995; Hibshoosh, Johnson, & Weinstein, 1991; O’Brien, Megosh, Gilliard, & Soler, 1997). We have not found evidence that increased polyamine intake can promote oncogenic transformation in normal untreated animals (Soda, Dobashi, Kano, Tsujinaka, & Konishi, 2009). A possible connection between polyamine intake and cancer mortality rate should be analyzed, as further studies are conducted to determine whether regular ingestion of polyamine provides substantial protection against CVD and help prolong human life.
